# Impact of COVID-19 on health care and quality of life in women with breast cancer

**DOI:** 10.1093/jncics/pkad033

**Published:** 2023-05-18

**Authors:** Charlotte Myers, Kathleen Bennett, Catherine Kelly, Janice Walshe, Nollaig O’Sullivan, Mary Quinn, Therese Lyons, Catherine Weadick, Caitriona Cahir

**Affiliations:** School of Population Health, Royal College of Surgeons Ireland, Dublin, Ireland; Data Science Centre, School of Population Health, Royal College of Surgeons Ireland, Dublin, Ireland; Department of Oncology, Mater Misericordiae University Hospital, Dublin, Ireland; Medical Oncology Department, St Vincent’s University Hospital, Dublin, Ireland; Medical Oncology Department, St Vincent’s University Hospital, Dublin, Ireland; Medical Oncology Department, St Vincent’s University Hospital, Dublin, Ireland; Medical Oncology Department, St Vincent’s University Hospital, Dublin, Ireland; Department of Oncology, Mater Misericordiae University Hospital, Dublin, Ireland; Data Science Centre, School of Population Health, Royal College of Surgeons Ireland, Dublin, Ireland

## Abstract

**Background:**

The aims of this study were to explore the impact of COVID-19 on health-care services and quality of life (QoL) in women diagnosed with breast cancer (BC) in Ireland and whether the impact varied by social determinants of health (SDH).

**Methods:**

Women diagnosed with BC completed a questionnaire measuring the impact of COVID-19, disruption to BC services, QoL, SDH, and clinical covariates during COVID-19 restrictions. The association between COVID-19 impact and disruption to BC services and QoL was assessed using multivariable regression with adjustment for SDH and clinical covariates. An interaction between COVID-19 impact and health insurance status was assessed within the regression models.

**Results:**

A total of 30.5% (n = 109) of women reported high COVID-19 impact, and these women experienced more disruption in BC services (odds ratio = 4.95, 95% confidence interval = 2.28 to 10.7, *P* < .001) and lower QoL (β = −12.01, SE = 3.37, *P* < .001) compared with women who reported low COVID-19 impact. Health insurance status moderated the effect of COVID-19 on disruption to BC services and QoL. Women who reported high COVID-19 impact experienced more disruption to BC services and lower QoL compared with women with low COVID-19 impact; however, the magnitude of these unfavorable effects differed by insurance status (*P*_interaction_  < .05).

**Conclusions:**

There was a large disruption to BC services and decrease in QoL for women with BC in Ireland during the pandemic. However, the impact was not the same for all women. It is important that women with BC are reintegrated into proper care and QoL is addressed through multidisciplinary support services.

The COVID-19 pandemic has had a global public health impact (1). Since the start of the COVID-19 pandemic, health services for cancer in most countries have been disrupted (2), and recovery from such disruptions, including screening and treatment, has generally been slow (3). Breast cancer (BC) is one of the most common cancers, and women with a diagnosis of BC were classified as vulnerable during the pandemic because of their compromised health status (4). To reduce the risk of COVID-19 for BC patients, diagnostic and surgical procedures were delayed, treatment plans changed, and routine follow-up visits postponed (5-7). Across several countries, studies have shown that many BC patients experienced a change in their recommended treatment during the pandemic (8,9).

There is a lack of evidence quantifying various COVID-19 stressors specific to women with BC, including worries, concerns, and experiences related to the pandemic. However, there is evidence that delays and changes in BC treatments because of the pandemic have had a negative impact on women’s emotional well-being, including concerns about cancer prognosis and fear of recurrence (10). BC patients and survivors have reported high rates of anxiety, depression, distress, and loneliness during this time (11,12), yet few studies have assessed the impact of COVID-19 stressors on women’s quality of life (QoL), including their physical, functional, emotional, and social well-being, such as lymphedema, pain, and burnout (7).

Evidence is emerging of the potential health inequalities pertaining to COVID-19 (13). Interactions between risk factors and comorbidities with socioeconomic circumstances (eg, social determinants of health [SDH]) can increase the likelihood of an individual contracting COVID-19; the interaction can also affect their overall health and well-being (13,14). The SDH framework can be used to describe disease occurrence, distribution, and consequences, and it can be applied to varying contexts to better understand health disparities (15,16). Patterns of social disparities (eg, socioeconomic status, education, employment) may contribute to a greater disruption of health services for certain individuals, which may also result in negative health outcomes, especially during health crises such as COVID-19 (13,17).

Studies on health inequalities pertaining to COVID-19 and BC are limited and include SDH such as age, insurance status, race, and income (9,18-20). Specifically in Ireland, previous research has associated health inequalities with health insurance status; individuals with private health insurance typically access services quicker than those without private insurance (21). Therefore, the aims of this study were to 1) explore the impact of COVID-19 on the disruption of health-care services and QoL, and 2) determine whether the impact of COVID-19 on the disruption of health-care services and QoL varies according to SDH (eg, health insurance) in women with BC in Ireland.

## Methods

### Study design and setting

This cross-sectional survey is the baseline data collection as part of a prospective explanatory sequential study (22). Baseline surveys were distributed starting at the end of September 2020 and completed in April 2021, which corresponds to the second and third waves of COVID-19 infection and government restrictions in Ireland (23). Ethical approval was obtained in June 2020 by the Office for National Research Ethics Committee in Ireland (20-NREC-COV-078).

### Participants

Women were eligible to participate if they had a diagnosis of BC within the past 5 years, were living in Ireland, were over 18 years of age, were English speaking, and had no known serious psychiatric conditions. Women were recruited through a social media campaign, and surveys were also distributed through BC centres via nonprobability sampling techniques (24). Informed consent was obtained by all participants included in the study, and data were self-reported.

### Outcome measures (disruption of BC services and QoL)

To understand the impact of COVID-19 on the disruption BC services, participants were categorized as binary (yes or no) for disrupted BC services, counting any cancellation and/or delay for BC services. QoL was measured with the Functional Assessment of Cancer Therapy-Breast (FACT-B), which includes physical well-being, social well-being, emotional well-being, functional well-being, and an additional BC-specific subscale. Overall scores range from 0 to 148, and higher scores indicate increased QoL (25).

### Impact of COVID-19

The impact of COVID-19 was assessed using the COVID-19 Stressors Questionnaire, which includes 14 items on daily life, workplace, financial stability, treatment changes, and health-related concerns specific to BC and COVID-19. The degree of concern is measured with a 5-point Likert scale, and the total score is calculated by averaging the level of concern for all stressors (26). For the disruption of BC services outcome, the COVID-19 Stressors Questionnaire was amended to exclude 2 questions related to BC treatment disruption. Low COVID-19 impact equated to an average level of concern less than 1, and high COVID-19 impact equated to an average level of concern of at least 1.

### SDH covariates

The following self-reported SDH were measured: age, education level, health insurance, region, employment status, and marital status. Age was included as an SDH on the basis of recently published literature, which highlights age-based health inequities during the COVID-19 pandemic, in particular the vulnerability of older people regarding access to health care and lack of mental health protection in younger populations (27). For age, participants were categorized as younger than 60 years vs 60 years and older. For education level, participants were categorized as low (primary or secondary) education vs high (third-level or postgraduate) education. For health insurance, participants were categorized as with or without private insurance. For region, participants were categorized as urban (living in a city or town) vs rural (living in a village or countryside). For employment status, participants were categorized as employed, unemployed (eg, unable to work, sickness, disability, student, or unemployed), or retired or looking after family and home. For marital status, participants were categorized either as married or in a long-term relationship or as not in a relationship.

### Clinical covariates

The following clinical covariates were measured: time since BC diagnosis, treatment status, cancer stage at diagnosis, and comorbidities. For time since diagnosis, participants were categorized as within the year, 1-2 years postdiagnosis, or 3-5 years postdiagnosis. For treatment status, participants were categorized as receiving active treatment (eg, surgery, chemotherapy, and/or radiation therapy) vs postactive treatment. For cancer stage at diagnosis, participants were categorized as early-stage (stage I-II) vs late-stage (stage III-IV) diagnosis. Participants were categorized as having 1 or no comorbidities vs 2 or more comorbidities.

### Study size

The proposed sample size for completed surveys was calculated to be n = 385, providing precision of at least ±5% on a prevalence of 50% for any response. To allow for incomplete surveys, n = 500 was the target study size for those responding to the survey.

### Statistical analysis

Descriptive statistics including frequencies (percentages) for categorical data and means (SD) for continuous data were calculated for outcome variables (disruption of BC services and QoL), COVID-19 impact, and all SDH and clinical covariates. COVID-19 impact differences between SDH and clinical covariates were examined using χ^2^ tests. Multivariable logistic and linear regression models were used to examine the association between COVID-19 impact and disrupted BC services and QoL with adjustment for SDH and clinical covariates. Adjusted odds ratios (ORs) and 95% confidence intervals (CIs) are presented for disrupted BC services, and adjusted regression coefficients (*β*) and standard errors (SE) are presented for QoL. To establish if SDH (eg, health insurance) moderates the impact of COVID-19 on BC service disruption and QoL, an interaction between COVID-19 impact and health insurance status was assessed within each multivariable regression model. All missing data were omitted from analyses, and 2-sided *P* values less than .05 were considered statistically significant. The data were analyzed using Stata Version 16.1.

## Results

### Study population

A total of n = 387 participants completed the survey; 162 participants completed the online survey, and 225 participants completed the survey through their BC clinic. A total of 357 (92%) women completed the entire COVID-19 Stressors Questionnaire and were included in the analysis. There were some differences between those who completed the entire COVID-19 impact questionnaire compared with those who did not. Those who did not complete the questionnaire (n = 30) were older, less educated, without private insurance, retired, and further removed from their cancer diagnosis (*P* < .05).

The average age of women was 53.6 years (SD = 10.2) The majority had an ethnicity of Irish (92.5%), were highly educated (75.9%) and either employed (46.8%) or retired from employment (26.2%), and over one-half (63.7%) of the women reported having private health insurance. All 26 counties in the Republic of Ireland were represented in the study, with County Dublin representing close to one-half of the study population (40.0%), which aligns with the geographical distribution of BC incidence in Ireland (28). At survey completion, women reported their time since diagnosis to be within the year (40.0%), 1-2 years prior (19.7%), or 3-5 years prior (40.3%). Most women reported an early-stage diagnosis (stage I or II) (70.4%) and reported to be post active treatment (72.8%) for BC. Additionally, most women (81.7%) reported having 1 or no comorbidities.

### Impact of COVID-19

Table 1 presents the SDH and clinical characteristics of women by COVID-19 impact (n = 357). Approximately 30.5% (n = 109) of women experienced a high COVID-19 impact. A higher proportion of women who reported a high COVID-19 impact were younger than 60 years (*P* = .03), were living in an urban region (*P* = .01), were employed (*P* = .02), and had 2 or more comorbidities (*P* = .02) compared with those who reported a low COVID-19 impact (*P* = .01). A higher proportion of women who reported a low COVID-19 impact were retired (*P* = .02) compared with those who reported a high COVID-19 impact.

**Table 1.** Social determinants of health and clinical characteristics of women with breast cancer experiencing a low vs high COVID-19 impact (N = 357)

	Low COVID impact (n = 248)	High COVID impact (n = 109)	*P* ^b^
	N (%)^a^	N (%)^a^	
Age			.03^b^
Under 60	168 (70.0)	85 (81.0)	
60 and older	72 (30.0)	20 (19.1)	
Region			.01^b^
Urban	141 (58.8)	77 (73.3)	
Rural	99 (41.2)	28 (26.7)	
Education			.84
Low	57 (23.7)	26 (24.8)	
High	183 (76.3)	79 (75.2)	
Employment			.02^b^
Employed	102 (42.5)	59 (56.7)	
Unemployed	66 (27.5)	27 (26.0)	
Retired	72 (30.0)	18 (17.3)	
Relationship status			.42
Married/in a relationship	177 (73.4)	72 (69.2)	
Not in a relationship	64 (26.6)	32 (30.8)	
Insurance			.30
Private insurance	157 (65.4)	62 (59.6)	
No private insurance	83 (34.6)	42 (40.4)	
Cancer stage at diagnosis			.95
Early (stage I-II)	165 (70.5)	73 (70.2)	
Late (stage III-IV)	69 (29.5)	31 (29.8)	
Diagnostic cohort			.76
Active treatment	66 (26.7)	30 (28.3)	
Postactive treatment	181 (73.3)	76 (71.7)	
Comorbidities			.02^b^
1 or less	199 (85.0)	77 (74.0)	
2 or more	35 (15.0)	27 (26.0)	
Time since cancer diagnosis			0.92
Within the year	95 (40.4)	41 (39.1)	
1-2 y	45 (19.2)	22 (20.9)	
3-5 y	95 (40.4)	42 (40.0)	

a Count and percentage among nonmissing responses. Missing data for each variable used in analysis include age: n = 12; region: n = 12; education: n = 12; employment: n = 13; relationship status: n = 12; insurance status: n = 13; cancer stage: n = 19; treatment status: n = 4; comorbidities: n = 19; time since diagnosis: n = 17.

b *P* < .05.

### Disruption of BC services

Table 2 presents the unadjusted and adjusted association between COVID-19 impact and disrupted BC services after adjusting for SDH and clinical covariates and including the interaction effect (n = 282). Most women (n = 185, 53.9%) experienced disruption to their BC services during the pandemic. In the unadjusted analyses, women who reported a high COVID-19 impact were statistically significantly more likely to experience a disruption to their BC services. No SDH were statistically significantly associated with disrupted BC services. In terms of clinical covariates, women in postactive treatment and further removed (1-2 years and 3-5 years) from their initial BC diagnosis reported more disrupted BC services (*P* < .05). With multivariable analyses, there remained a difference in disruption of BC services between women who reported a high COVID-19 impact vs low COVID-19 impact (adjusted OR = 4.95, 95% CI = 2.28 to 10.7, *P* < .001). Additionally, clinical covariates such as treatment status (adjusted OR = 3.67, 95% CI = 1.76 to 7.69, *P* = .001) and time since BC diagnosis (adjusted OR = 2.53, 95% CI = 1.15 to 5.56, *P* = .02; adjusted OR = 4.07, 95% CI = 2.04 to 8.10, *P* < .001) remained associated with disrupted BC services.

**Table 2.** Unadjusted and adjusted odds ratios and 95% confidence intervals for disrupted breast cancer (BC) services by COVID-19 impact and social determinants of health and clinical characteristics of women with BC (N = 282)^b^

Characteristic	Disrupted BC services	Nondisrupted BC services	Unadjusted OR (95% CI)	Adjusted OR (95% CI)^b^
	N (%)^a^	N (%)^a^		
COVID impact				
Low	101 (54.6)	113 (71.5)		
High	84 (45.4)	45 (28.5)	2.09 (1.33 to 3.28)^c^	4.95 (2.28 to 10.7)^c^
Age, y				
<60	137 (76.5)	110 (71.9)		
≥60	42 (23.5)	43 (28.1)	0.78 (0.49 to 1.28)	0.67 (0.31 to 1.49)
Region				
Urban	113 (63.1)	93 (61.2)		
Rural	66 (36.9)	59 (38.8)	0.92 (0.59 to 1.44)	1.15 (0.65 to 2.06)
Education				
Low	41 (22.9)	39 (25.5)		
High	138 (77.1)	114 (74.5)	1.15 (0.70 to 1.91)	1.37 (0.68 to 2.74)
Employment				
Employed	90 (50.6)	65 (42.7)		
Unemployed	42 (23.6)	48 (31.6)	0.63 (0.37 to 1.07)	0.67 (0.35 to 1.30)
Retired	46 (25.8)	39 (25.7)	0.85 (0.50 to 1.45)	1.21 (0.55 to 2.65)
Relationship status				
Married/in relationship	129 (72.9)	109 (70.8)		
Not in relationship	48 (27.1)	45 (29.2)	0.90 (0.56 to 1.46)	0.90 (0.48 to 1.71)
Insurance				
Private	106 (59.9)	105 (68.6)		
No private	71 (40.1)	48 (31.4)	1.46 (0.93 to 2.31)	1.38 (0.64 to 2.95)
Cancer stage at diagnosis				
Early (stage I-II)	128 (72.3)	102 (68.9)		
Late (stage III-IV)	49 (27.7)	46 (31.1)	0.85 (0.53 to 1.37)	1.00 (0.53 to 1.86)
Treatment status				
Active	22 (12.0)	70 (44.9)		
Postactive	161 (88.0)	86 (55.1)	5.96 (3.45 to 10.3)^c^	3.67 (1.76 to 7.69)^c^
Comorbidities				
≤1	139 (79.0)	127 (85.2)		
≥2	37 (21.0)	22 (14.8)	1.54 (0.86 to 2.74)	1.21 (0.56 to 2.64)
Time since cancer diagnosis				
Within 1 y	44 (25.0)	87 (57.6)		
1-2 y	40 (22.7)	24 (15.9)	3.30 (1.77 to 6.14)^c^	2.53 (1.15 to 5.56)^c^
3-5 y	92 (52.3)	40 (26.5)	4.55 (2.71 to 7.64)^c^	4.07 (2.04 to 8.10)^c^

a Count and percentage among nonmissing responses. Missing data for each variable used in analysis include disrupted BC care: n = 14; age: n = 12; region: n = 12; education: n = 12; employment: n = 13; relationship status: n = 12; insurance status: n = 13; cancer stage: n = 19; treatment status: n = 4; comorbidities: n = 19; time since diagnosis: n = 17. CI = confidence interval; OR = odds ratio.

b Number of observations used for the adjusted regression model was N = 282.

c *P* < .05.

**Figure 1.**
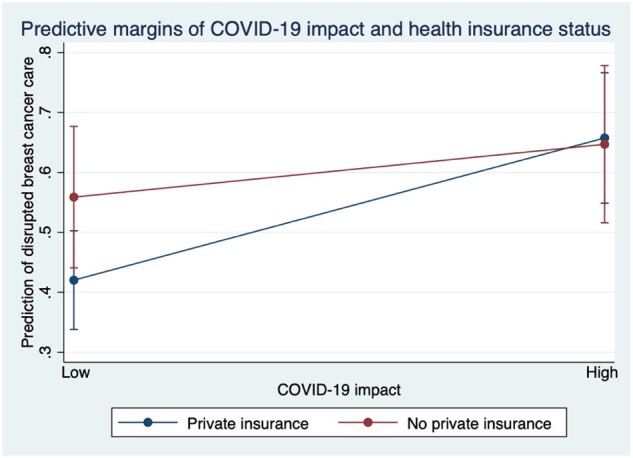
Margins plot for the interaction between COVID-19 impact and health insurance status on disrupted breast cancer services with 95% confidence intervals.

Not having private health insurance vs having private health insurance was found to moderate the impact of COVID-19 on disruption to BC services (*P* < .05). Women who reported a high COVID-19 impact and private insurance (OR = 2.65, 95% CI = 1.47 to 4.78, *P* = .001) and a high COVID-19 impact and no private insurance (OR = 2.53, 95% CI = 1.30 to 4.92, *P* = .006) experienced more disruption to BC services compared with women with a low COVID-19 impact and private insurance. The margins plot for the interaction between COVID-19 impact and health insurance on disruption to BC services (Figure 1) shows a negative effect of COVID-19 impact for private health insurance. A high COVID-19 impact leads to greater disruption in BC services for those with private insurance compared with a low COVID-19 impact. There is also a negative effect of COVID-19 impact for no private insurance. There is an increase in disruption on BC services for a high COVID-19 impact compared with a low COVID-19 impact, but the increase is less for women without private health insurance compared with those with private health insurance.

### QoL

**Figure 2.**
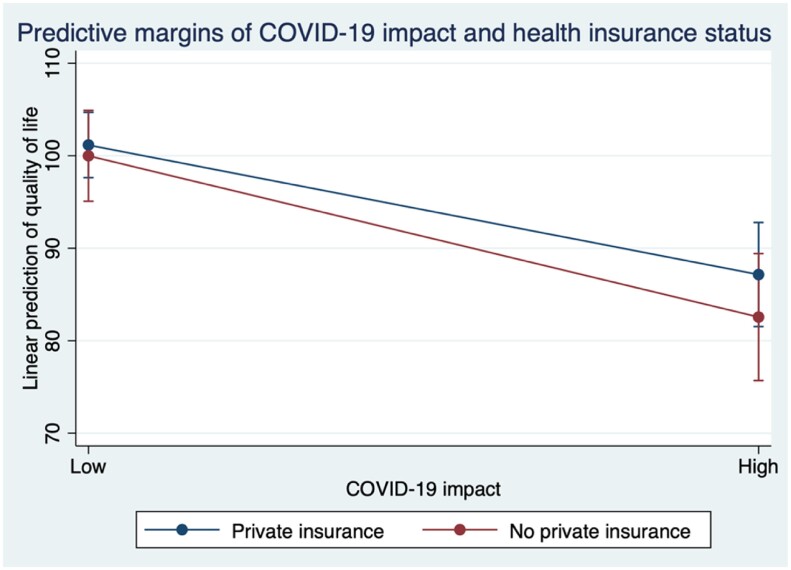
Margins plot for the interaction between COVID-19 impact and health insurance status on quality of life with 95% confidence intervals.

Table 3 presents the unadjusted and adjusted association between COVID-19 impact and QoL after adjustment for SDH and clinical characteristics and including the interaction effect (n = 287). The average overall score for health-related QoL was 96.1 (SD = 23.3). In the unadjusted analyses, women who reported high COVID-19 impact also reported statistically significantly lower QoL. For SDH, women younger than 60 years of age and women with unemployment status reported statistically significantly lower QoL. Additionally, women receiving active BC treatment during the pandemic and women with 2 or more comorbidities reported lower QoL (*P* < .05). In multivariable analyses, a high COVID-19 impact remained associated with lower QoL scores (β = −12.0, SE = 3.37, *P* < .001). For SDH, older age was associated with a higher QoL (β = 7.75, SE = 3.64, *P* = .034), and unemployment remained associated with lower QoL (β = −14.61, SE = 3.07, *P* < .001). For clinical covariates, having 2 or more comorbidities remained associated with a lower QoL (β = −8.82, SE = 3.45, *P* = .01). Having private health insurance vs not having private health insurance was found to moderate the impact of COVID-19 on QoL (*P* < .05). Compared with women with a low COVID-19 impact and private insurance, women with a high COVID-19 impact and private insurance (β = −14.01, SE = 3.38, *P* < .001) and a high COVID-19 impact and no private insurance (β = −18.6, SE = 3.92, *P* < .001) experienced a lower QoL. The margins plot for the interaction between COVID-19 impact and health insurance on health-related QoL (Figure 2) shows a negative effect of COVID-19 impact for both private health insurance and no private health insurance. A high COVID-19 impact lowered QoL for both those with and without private health insurance compared with a low COVID-19 impact, and the decrease in QoL was greater for women without private insurance.

**Table 3.** Unadjusted and adjusted associations for quality of life (QoL) by COVID-19 impact and social determinants of health and clinical characteristics of women with breast cancer (N = 287)^a^

Characteristic	FACT-B	Unadjusted β (SE)	Adjusted β (SE)^a^
	Mean (SD)^b^		
COVID impact			
Low	100.8 (22.5)		
High	85.6 (21.4)	−15.27 (2.59)^c^	−12.0 (3.37)^c^
Age, y			
<60	93.5 (22.4)		
≥60	102.9 (24.4)	9.39 (2.87)^c^	7.75 (3.64)^c^
Region			
Urban	95.3 (23.3)		
Rural	97.8 (23.4)	2.46 (2.63)	2.67 (2.65)
Education			
Low	95.2 (27.1)		
High	96.3 (22.2)	1.06 (3.03)	3.16 (3.30)
Employment			
Employed	98.5 (22.8)		
Unemployed	85.3 (20.6)	−13.20 (2.93)^c^	−14.6 (3.07)^c^
Retired	103.1 (23.4)	4.63 (2.98)	−0.64 (3.64)
Relationship status			
Married/in relationship	97.5 (22.1)		
Not in relationship	92.6 (26.3)	−4.89 (2.83)	−2.10 (2.90)
Insurance			
Private	97.2 (21.9)		
No private	94.1 (25.9)	−3.12 (2.65)	0.69 (3.44)
Cancer stage at diagnosis			
Early (stage I-II)	97.8 (24.0)		
Late (stage III-IV)	92.4 (21.0)	−5.34 (2.80)	−2.20 (2.82)
Treatment status			
Active	91.4 (20.8)		
Postactive	97.8 (23.9)	6.47 (2.80)^c^	5.46 (3.40)
Comorbidities			
1 or less	97.5 (22.8)		
2 or more	88.2 (25.2)	−9.30 (3.30)^c^	−8.82 (3.45)^c^
Time since cancer diagnosis			
Within 1 y	95.2 (21.3)		
1-2 y	92.6 (23.1)	−2.58 (3.48)	−3.05 (3.83)
3-5 y	99.3 (24.9)	4.11 (2.85)	1.37 (3.23)

a Number of observations used for the adjusted regression model was N = 287.

b Mean (SD) among nonmissing responses. Missing data for each variable used in analysis include QoL: n = 11; age: n = 12 ; region: n = 12; education: n = 12; employment: n = 13; relationship status: n = 12; insurance status: n = 13; cancer stage: n = 19; treatment status: n = 4; comorbidities: n = 19; time since diagnosis: n = 17. SE = standard error.

c *P* < .05.

## Discussion

This study has found that women living with and beyond BC have been statistically significantly affected by the COVID-19 pandemic. One-third of women reported experiencing a high impact of COVID-19 stressors on their daily lives, and those with a high COVID-19 impact experienced more disruption to BC services and lower QoL. However, the impact of COVID-19 was not the same for all women with BC. Younger women, women living in urban regions, and women with multi-morbidities reported a higher COVID-19 impact, whereas retired women reported a lower COVID-19 impact. Women in postactive treatment and women further removed from their initial BC diagnosis reported statistically significantly more disrupted BC services. Younger women, unemployed women, and women with multi-morbidities reported a statistically significantly lower QoL. Lastly, health insurance was found to moderate the impact of COVID-19 statistically significantly on both disruption to BC services and QoL.

Globally, there has been a large disruption to BC services during the pandemic (29), and this study coincides with this trend. Previous research found that follow-up BC visits decreased by approximately one-half from 2019 to 2020 (9). The existing literature is inconsistent in findings on disruption for active BC treatment, and findings are conditional on the type of BC treatment received (30-32). For example, one study found that the number of BC surgeries declined during the pandemic (30), whereas another found that more surgical procedures were performed during the pandemic and the waiting time to surgery was less than prepandemic times (31). In addition, one study found that initiation of radiation treatment was lower in 2020 compared with 2019 (33), whereas another found that the delays during the pandemic for radiation treatment did not differ statistically significantly from prepandemic times (32). In comparison, our study found that women who were diagnosed within 2020 and/or received active treatment during the pandemic reported less disrupted BC services, suggesting prioritization based on need.

The minimal important difference for interpreting group differences or changes in QoL over time using the FACT-B is estimated to be in the range of 3-8 points (34), and our findings suggests a large negative impact of COVID-19 on QoL compared with other studies conducted before the pandemic. For example, Al-Kaylani et al. (35) found the average FACT-B score among women with BC to be 108.5, which is more than 10 points higher than the average result in our study.

Research is limited on QoL during the pandemic; however, physical, social, emotional, and cognitive functioning have been shown to be common concerns for women with BC (7,36). One study found that women reported lower QoL with increased financial problems such as unemployment due to the pandemic (19), which is consistent with our study; unemployed women reported statistically significantly lower QoL than women who were employed or retired.

For both outcomes (disruption to BC services and QoL), the interaction between COVID-19 impact and health insurance was statistically significant. For both categories of COVID-19 impact, women without private health insurance reported statistically significantly lower QoL than women with private insurance. Women with private insurance and a low COVID-19 impact reported the highest QoL, whereas women without private insurance and a high COVID-19 impact reported the lowest QoL. Therefore, the interaction between COVID-19 impact and health insurance status is likely a determinant for QoL for women in our study. There is limited related research conducted during the pandemic; however, one study in the United States found health insurance status to be associated with financial well-being for BC patients (19).

The interaction between COVID-19 impact and health insurance was different for disruption to BC services. Not having private insurance was associated with greater disruption to BC services when COVID-19 impact was low; however, when COVID-19 impact was high, having private health insurance was associated with slightly higher disruption compared with not having private insurance. Ireland remains one of the only European countries that does not provide universal health care to its citizens (37). The Irish health system operates on a complex 2-tiered, public and private basis; nearly one-half of the population opt for private insurance, which primarily provides more rapid access to health care through private and/or public secondary and tertiary specialist services (38,39). During the pandemic, private hospitals in Ireland were used as public hospitals to provide additional hospital capacity to deal with the impact of the pandemic (37); this may explain the negative effect for women with private insurance and a high COVID-19 impact on BC service disruption. Comparably, one study conducted in the United States found that women with limited ability to pay (ie, public health insurance) were 3 times more likely to experience treatment delays (18), yet this study did not assess the interaction with COVID-19 impact.

This study makes an important contribution to limited evidence of the impact of COVID-19 on women with BC in Ireland. The study provides descriptive data on the impact of the pandemic, disruption of BC services, and health-related QoL. Moreover, this study is one of few studies to consider varied experience by SDH. However, limitations include the potential for bias related to external validity or generalizability. A high percentage of the study population was highly educated, and they were also slightly younger than a previous population study of QoL in women with BC (40). We aimed to make the sample representative of the general BC population by using 2 different recruitment methods to help reduce this bias: a social media campaign and a paper-based survey distribution through BC clinics. Participants were able to save and return to the online survey if they preferred, allowing higher completion rates.

In conclusion, the study identifies the impact of the pandemic for women living with and beyond BC in Ireland, a country that experienced severe restrictions and longer lockdowns compared with other countries (41). There has been a large disruption to BC services during the pandemic, including active treatment and postactive treatment, and this impact, along with a decrease in QoL, was not the same experience for all women. It is important to identify the women who experienced a larger disruption to BC services so they can be reintegrated into proper BC care along the entire cancer continuum. Personalized BC care can ensure equality in BC care by considering both biological and clinical characteristics along with nonbiological characteristics, such as the SDH (42). Furthermore, BC services should involve the multi-disciplinary needs of women to improve QoL, such as psychosocial interventions. Health-care providers should address these needs and provide more guidance through communication, resources, and support organizations (19). Web-based (eg, telemedicine) psychosocial approaches have shown to be beneficial (43), and women with BC were not only satisfied with telemedicine during the pandemic but found the remote communication with their BC care team to improve anxiety levels (44). Follow-up research on QoL is needed to evaluate whether women’s well-being has improved since the government lockdown; additional research is also needed to explore the high impact of COVID-19 in greater detail.

## Data Availability

The data underlying this article cannot be shared due to the privacy of individuals that participated in the study. Additional summary level data without individual data can be provided.
